# Characteristics and Risk Factors Associated With Mortality in Critically Ill Patients With COVID-19

**DOI:** 10.7759/cureus.14442

**Published:** 2021-04-12

**Authors:** Yannick Vogels, Sjaak Pouwels, Jos van Oers, Dharmanand Ramnarain

**Affiliations:** 1 Intensive Care Medicine, Elisabeth-TweeSteden Hospital, Tilburg, NLD

**Keywords:** sars-cov-2 (severe acute respiratory syndrome coronavirus -2), morbidity and mortality, pulmonology and critical care

## Abstract

Purpose

To describe clinical characteristics and outcomes of ICU patients with COVID-19 and to investigate differences between survivors and non-survivors.

Methods

Demographics, symptoms, laboratory values, comorbidities and outcomes were extracted retrospectively from the medical records of ICU patients with confirmed COVID-19 pneumonia from the Elisabeth-TweeSteden Hospital in Tilburg, the Netherlands from March until June 2020. Primary outcome was 28-day mortality and secondary outcomes were differences between survivors and non-survivors.

Results

Between March 1 and June 4, 2020, 114 patients with COVID-19 were admitted to the ICU. There were 83 (72.8%) survivors and 31 (27.2%) non-survivors. Non-survivors were significantly older (72.0 years [interquartile range, IQR 67.0-76.0] versus 65.0 years [IQR 58.0-73.0], P* *= 0.002), had a significantly higher Acute Physiology And Chronic Health Evaluation (APACHE) score (54 [IQR 45-72] versus 43 [IQR 36-53], P* *< 0.001) and Sequential Organ Failure Assessment (SOFA) score (7 [IQR 4-7] versus 5 [IQR 3-6], P = 004). cTnT values were significantly higher in non-survivors due to more myocarditis (83.9% versus 40.8%, P* *< 0.001). A multivariate Cox regression model revealed SOFA score (hazard ratio, HR 1.337, 95% CI 1.131-1.582, P* *= 0.001) to be an independent predictor of 28-day mortality.

Conclusion

We demonstrated a 28-day mortality rate of 27.2% in our cohort. These patients were older and presented with a higher severity of illness and more organ failure.

## Introduction

In late 2019, the novel coronavirus infectious disease 2019 (COVID-19) caused by severe acute respiratory syndrome coronavirus 2 (SARS-CoV-2) infection began to spread in China, causing a global pandemic in the first few months of 2020. As of January 18, 2021, 93,805,612 SARS-CoV-2 infections and 2,026,093 COVID-19-related deaths had been reported worldwide [1]. On February 27, 2020, the first confirmed COVID-19 case in the Netherlands was admitted to the Elisabeth-TweeSteden Hospital (ETZ) in Tilburg. In the following weeks, the number of confirmed cases admitted to the general wards and ICU of Dutch hospitals rose quickly.

Although the majority of COVID-19 infections progresses mildly, 5%-10% of cases develop severe symptoms, rapidly culminating in respiratory failure and/or multiple organ dysfunction and requiring ICU admission [2].

While investigating 3,988 COVID-19 confirmed patients admitted to the ICU in Lombardy, Italy, Graselli et al. found a mortality of 48.8% during ICU admission. In addition, chronic obstructive pulmonary disease (COPD), hypercholesterolemia and diabetes were found to be significantly associated with mortality [3]. Worldwide, other reports also found that the presence of various comorbidities in patients with COVID-19 is significantly associated with mortality [4-9].

In this report, we present the clinical characteristics and outcomes of critically ill patients admitted to the ICU in the ETZ with laboratory-confirmed COVID-19 infection. We aim to identify risk factors for in-hospital mortality in this patient population and to explore additional differences between survivors and non-survivors.

## Materials and methods

Study design and setting

We performed a retrospective cohort study at the ICU in the ETZ. This study was performed in accordance with the ethical standards as laid down in the 1964 Declaration of Helsinki and was conducted according to the STrengthening the Reporting of Observational studies in Epidemiology (STROBE) statement [10]. The study protocol was reviewed and approved by the local Institutional Review Board (IRB); the requirement for written informed consent was waived by the IRB due to the retrospective and observational design of the study (protocol number: L0977.2020).

Participants

Subjects eligible for inclusion were patients with laboratory-confirmed COVID-19 infection, who were admitted to the ICU in the ETZ between March 1 and June 4, 2020. Patients who were transferred to other ICUs during their admission were also included. All included patients were diagnosed according to the WHO criteria [11]. Patients were suspected of COVID-19 when the following symptoms were present: dyspnoea, increased respiratory insufficiency, decreased blood oxygen saturation and the need for supportive oxygen therapy [11]. As diagnostic modality, a conventional chest X-ray or a CT scan was used [11].

Reverse transcriptase-polymerase chain reaction assay (RT-PCR) was done on throat swab samples to confirm the diagnosis. In the case of high clinical suspicion and multiple negative tests (or imaging), deep bronchial aspirates and faeces tests were performed to determine the diagnosis [12,13]. Patients with pneumonia were excluded if no SARS-CoV-2 could be detected by RT-PCR.

Variables and outcomes

Patient characteristics and information about outcome were extracted from the electronic medical records of included patients. This included demographics, severity of illness, laboratory tests and signs and symptoms at ICU admission, as well as the need for life-sustaining treatment or use of pharmacological therapy during ICU stay. Laboratory tests included complete blood count, hepatic and renal function tests, arterial blood gas, biomarkers of inflammation (C-reactive protein [CRP], ferritin) and high sensitive cardiac troponin T (hs-cTnT). We determined the severity of illness using the Acute Physiology And Chronic Health Evaluation IV (APACHE IV) score. Organ dysfunction was evaluated on day 1 by applying the Sequential Organ Failure Assessment (SOFA) score in conjunction with laboratory tests [14]. Cardiac injury was defined as serum hs-cTnT above the upper limit of the reference range (>0.014 µg/L). Pulmonary embolus (PE) was diagnosed using CT. Deep venous thrombosis (DVT) was diagnosed using ultrasonography.

The primary outcome was defined as 28-day mortality after ICU admission. Secondary outcomes were defined as ICU length of stay and the use of life-sustaining treatment, such as invasive mechanical ventilation (IMV), continuous renal replacement therapy (CRRT) and vasopressor use.

Statistical analysis

Categorical variables were compared using χ2 or Fisher exact tests. In addition, counts and proportions were tabulated for categorical variables. Continuous variables were compared with the Student’s t-test or Mann-Whitney U-test and were presented as the mean ± standard deviation (SD) or median (interquartile range [IQR]) as appropriate. Missing data of key variables are summarized in Table 5 (Appendix).

We constructed two multivariate models to determine the risk factors of 28-day mortality.

The first model was a univariate and multivariate Cox regression proportional-hazards model of risk factors associated with 28-day mortality in COVID-19 patients. Risk factors included in this model were the significant differences between our survivor and non-survivor groups as depicted in Table 1 and Table 2. The second model was constructed using backward selection of risk factors associated with 28-day mortality in COVID-19 patients. Backward selections were done by a step-by-step removal of non-significant variables until a minimum of five variables were left in the multivariate model [15]. If collinearity was suspected, one of both variables was chosen to enter into the Cox regression models by author consensus (YV and SP).

Variables present on ICU admission with P < 0.05 in univariate analysis or determined as risk factors of mortality in previous studies were entered into the model.

Differences with P < 0.05 were considered statistically significant. All reported P values are two-sided. Statistical analyses were performed using IBM SPSS Statistics version 25.0 for Windows software (SPSS Inc., Chicago, IL).

## Results

Population characteristics

**Figure 1:**
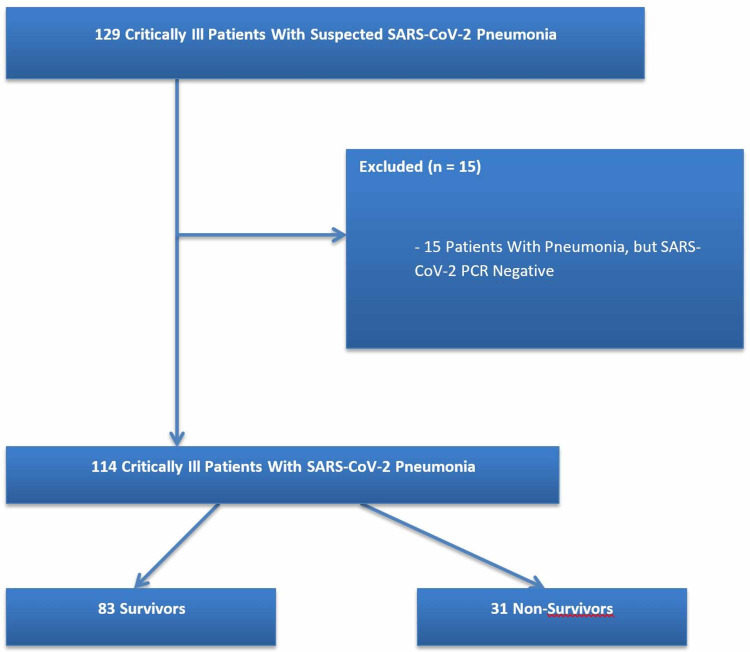
Patient inclusion flow diagram. Abbreviations: PCR, polymerase chain reaction; SARS-CoV-2, severe acute respiratory syndrome coronavirus 2.

A total of 129 patients who were suspected of COVID-19 infection were admitted to the ICU in the aforementioned study period. In 114 patients with pneumonia, COVID-19 was confirmed by RT-PCR. Figure 1 shows the study flow diagram of patients included in this study.

 

Table 1 provides an overview of the characteristics of included patients. Median age was 68.0 years (IQR 59.0-73.3), the majority of the included patients were male (76.3%). The 28-day all-cause mortality was 27.2% (31 of 114 patients). Comorbidities were present in 79 patients (69.3%), of which hypertension and diabetes mellitus type II were the most prevalent (28.1% and 25.4%, respectively). Regarding symptoms, cough, fever and dyspnoea were the most prevalent (94.7%, 77.2% and 82.5%, respectively). During the study period, the majority of the patients received treatment with hydroxychloroquine (69.3%), which is nowadays removed from our hospital’s protocol, due to lack of efficacy and evidence. In addition, 34 (29.8%) of the included patients received simultaneous treatment with lopinavir/ritonavir and hydroxychloroquine. Six patients (5.2%) received treatment with methylprednisolone.

**Table 1:** Characteristics of patients with COVID-19. Abbreviations: APACHE, Acute Physiological and Chronic Health Evaluation; BMI, body mass index; CRP, C-reactive protein; hs-cTnT, high-sensitivity cardiac troponin T; SOFA, Sequential Organ Failure Assessment; WBC, white blood cell; IQR, interquartile range; COPD, chronic obstructive pulmonary disease. *Bold values indicate significance. ^†^Variables expressed as median (interquartile range).

Characteristic	All (N = 114)	Survivor (N = 83)	Non-survivor (N = 31)	P-value^*^
Age, median (IQR)	68.0 (59.0-73.3)	65.0 (58.0-73.0)	72.0 (67.0-76.0)	0.002
Male, n (%)	87 (76.3)	62 (80.6)	25 (80.6)	0.62
BMI, kg/m^2^, median (IQR)	28.7 (25.9-32.7)	28.4 (26.0-32.4)	29.4 (25.7-33.8)	0.63
APACHE IV score on the first day of ICU admission, median (IQR)	47 (40-59)	43 (36-53)	54 (45-72)	<0.001
SOFA score on the first day of ICU admission, median (IQR)	5 (3-7)	5 (3-6)	7 (4-7)	0.004
Renal injury, n (%)	1 (0.9)	0 (0)	1 (3.2)	0.27
Liver injury, n (%)	0 (0.0)	0 (0)	0 (0)	NS
Myocardial injury, n (%)	62 (54.4)	42 (40.8)	26 (83.9)	<0.001
No. of comorbidities, median (IQR)	1 (0-2)	1 (0-2)	1 (1-2)	0.19
Any comorbidity, n (%)	79 (69.3)	66 (64.1)	28 (80.0)	0.12
Hypertension, n (%)	32 (28.1)	23 (27.7)	9 (29.0)	>0.99
COPD, n (%)	18 (15.8)	13 (15.7)	5 (16.1)	>0.99
Congestive heart failure, n (%)	19 (16.7)	12 (14.5)	7 (22.6)	0.40
Diabetes mellitus type II, n (%)	29 (25.4)	21 (25.3)	8 (25.8)	>0.99
Chronic renal failure, n (%)	6 (5.3)	3 (3.6)	3 (9.7)	0.34
Malignancy, n (%)	17 (14.9)	11 (13.3)	6 (19.4)	0.40
Stroke, n (%)	7 (6.1)	2 (2.4)	5 (16.1)	0.02
Immunosuppression, n (%)	8 (7.0)	7 (8.4)	1 (3.2)	0.44
Symptoms present at admission, n (%)				
Fever	88 (77.2)	62 (74.7)	26 (83.9)	0.45
Cough	108 (94.7)	79 (95.2)	29 (93.5)	0.66
Sputum	28 (24.6)	18 (21.7)	10 (32.3)	0.33
Dyspnoea	94 (82.5)	68 (81.9)	26 (83.9)	>0.99
Diarrhoea	21 (18.4)	18 (21.7)	3 (9.7)	0.18
Vomiting	15 (13.2)	12 (14.5)	3 (9.7)	0.76
Myalgia	19 (16.7)	15 (18.1)	4 (12.9)	0.59
Laboratory tests at admission^†^				
WBC, 10^9^/L	8.35 (6.10-11.30)	8.1 (6.0-11.3)	8.8 (6.6-11.3)	0.49
Lymphocytes, 10^9^/L	0.69 (0.49-0.94)	0.77 (0.54-0.96)	0.67 (0.43-0.90)	0.20
Neutrophils, 10^9^/L	5.93 (3.93-9.06)	5.88 (3.80-9.10)	6.49 (4.03-9.10)	0.66
Platelet count, 10^9^/L	228 (178-294)	229 (175-282)	220 (188-302)	0.98
CRP, mg/L	146.0 (89.8-204.5)	145.0 (90.0-194.0)	152.0 (89.0-236.0)	0.54
Bilirubin, µmol/L	7.0 (4.0-10.3)	5.5 (4.8-9.8)	8.0 (4.8-11.3)	0.11
Creatinine, µmol/L	79 (64-97)	76 (59-94)	93 (72-134)	0.001
Lactate, µmol/L	1.2 (0.9-1.6)	1.2 (0.8-1.5)	1.6 (1.1-2.0)	0.002
Ferritin, µg/L	1323.5 (720.8-2439.0)	1151.0 (585.5-1962.0)	1449.0 (1047.5-3603.5)	0.069
Blood D-dimer, µg/L	1375 (795-4152)	1279 (751-3406)	2076 (1164-13,398)	0.067
hs-cTnT, µg/L	0.018 (0.010-0.038)	0.013 (0.010-0.023)	0.032 (0.019-0.075)	<0.001
Medication, n (%)				
Hydroxychloroquine	79 (69.3)	59 (71.1)	20 (64.5)	0.50
Hydroxychloroquine and lopinavir/ritonavir	34 (29.8)	23 (27.7)	11 (35.5)	0.49
Methylprednisolone (Meduri)	6 (5.2)	3 (3.6)	3 (9.7)	0.34

Table 2 gives an overview of the clinical course and outcome of patients with COVID-19. In only eight patients (7.0%), high flow nasal oxygen (HFNO) was used. In total 88 patients (77.2%) were intubated and required invasive mechanical ventilation (IMV) at day 1 of the ICU admission. A total of 106 patients (93.0%) needed IMV during ICU stay. IMV duration varied between 9 and 25 days (median 14 days), prone position ventilation was required in 65 patients (57.0%) and the majority needed vasopressors (96 patients (84.2%)). Complications were noted in approximately one-third of the patients and included bacteraemia, deep venous thrombosis and pulmonary embolism. 

**Table 2:** Clinical course and outcome of COVID-19 patients. Abbreviations: CRRT, continuous renal replacement therapy; HFNC, high flow nasal oxygen; IMV, invasive mechanical ventilation; IQR, interquartile range. * Bold values indicate significance.

	All (N = 114)	Survivor (N = 83)	Non-survivor (N = 31)	P-value^*^
Duration of hospital admission before ICU admission, days, median (IQR)	1 (0-2)	1 (0-3)	0 (0-1)	0.001
Respiratory support at ICU admission				
HFNO, n (%)	8 (7.0)	6 (7.2)	2 (6.5)	>0.99
IMV (at day 1 of ICU admission), n (%)	88 (77.2)	61 (73.5)	27 (87.1)	0.14
Supportive care during ICU stay				
IMV, n (%)	106 (93.0)	77 (92.8)	29 (93.5)	>0.99
Duration of IMV, days, median (IQR)	14 (9-25)	16 (10-28)	9 (3-17)	<0.001
P_aO2_/F_IO2_ ratio, mmHg, median (IQR)	158.4 (116.1-194.3)	150.0 (114.4-199.4)	159.4 (127.5-176.3)	0.64
Prone ventilation during ICU stay, n (%)	65 (57.0)	47 (56.6)	18 (58.1)	>0.99
Vasopressor use, n (%)	96 (84.2)	68 (81.9)	28 (90.3)	0.39
CRRT, n (%)	9 (7.9)	4 (4.8)	5 (16.1)	0.060
Outcomes				
Complications during ICU stay				
Bacteraemia, n (%)	13 (11.4)	11 (13.3)	2 (6.5)	0.51
Pulmonary embolus, n (%)	18 (15.8)	12 (14.5)	6 (19.4)	0.57
Deep venous thrombosis, n (%)	10 (8.8)	9 (10.8)	1 (3.2)	0.28
ICU length of stay, days, median (IQR)	17 (10-32)	23 (13-35)	11 (3-18)	<0.001

Differences between survivors and non-survivors

Table 1 and Table 2 also show the differences between survivors and non-survivors. The non-survivors were significantly older (72.0 years (IQR 67.0-76.0) versus 65.0 years (IQR 58.0-73.0), P = 0.002), had a significantly higher APACHE IV score (54 (IQR 45-72) versus 43 (IQR 36-53), P < 0.001) and SOFA score (7 (IQR 4-7) versus 5 (IQR 3-6), P = 0.004). There was a significantly higher rate of myocardial damage among non-survivors (83.9% versus 40.8%, P < 0.001). With the exception of a higher prevalence of previous stroke in non-survivors (2 (2.4%) versus 5 (16.1%) patients, P = 0.02), there were no significant differences regarding the presence of comorbidities between survivors and non-survivors. In terms of laboratory test values at ICU admission, creatinine, lactate and hs-cTnT were all significantly higher in the non-survivor group. Due to early death, non-survivors had a significantly shorter IMV duration, ICU length of stay and a higher rate of CRRT.

Risk factors for mortality

Table 3 shows our first univariate and multivariate Cox regression proportional-hazards models of risk factors associated with 28-day mortality in critically ill COVID-19 patients. Age, APACHE-IV and SOFA score, presence of myocardial injury, previous stroke, creatinine, lactate and ICU length of stay were included in the model. Univariate analysis demonstrated significance for all variables. Therefore, they were included in a multivariate model. This resulted in only significance for SOFA score on the first day of ICU admission and ICU length of stay.

**Table 3:** Univariate and multivariate Cox regression proportional-hazards model of risk factors associated with 28-day mortality in COVID-19 patients. Abbreviations: APACHE, Acute Physiological and Chronic Health Evaluation; SOFA, Sequential Organ Failure Assessment; HR, hazard ratio. *Bold values indicate significance.

Variables	Univariate model			Multivariate model 1		
	HR	95% CI	P-value^*^	HR	95% CI	P-value^*^
Age	1.072	1.023-1.123	0.003	1.034	0.977-1.094	0.24
APACHE-IV (first day of ICU admission)	1.042	1.025-1.059	<0.001	1.013	0.991-1.035	0.24
SOFA (first day of ICU admission)	1.392	1.155-1.678	0.001	1.214	1.005-1.467	0.044
Myocardial injury	5.378	2.063-14.023	0.001	2.630	0.876-7.898	0.09
Previous stroke	3.528	1.346-9.250	0.01	1.824	0.624-5.328	0.27
Creatinine	1.003	1.002-1.005	<0.001	0.999	0.995-1.003	0.58
Lactate	1.161	1.017-1.326	0.03	1.099	0.949-1.272	0.21
ICU length of stay	0.895	0.853-0.939	<0.001	0.888	0.841-0.938	<0.001

Table 4 shows our second multivariate model using backward selection [15]. We arrived at this model after removing three non-significant covariates as explained by Altman et al. [15]. A higher SOFA score was found to be an independent predictor for 28­-day mortality in patients with COVID-19 admitted to the ICU (HR 1.337, 95% CI 1.131-1.582, P = 0.001).

**Table 4:** Univariate and multivariate Cox regression proportional-hazards model using backward selection of risk factors associated with 28-day mortality in COVID-19 patients. Abbreviations: APACHE, Acute Physiological and Chronic Health Evaluation; SOFA, Sequential Organ Failure Assessment; HR, hazard ratio. *Bold values indicate significance.

Variables	Multivariate model 1			Multivariate model 2		
	HR	95% CI	P-value^*^	HR	95% CI	P-value^*^
Age	1.034	0.977-1.094	0.24	1.046	0.995-1.100	0.08
APACHE-IV (first day of ICU admission)	1.013	0.991-1.035	0.24			
SOFA (first day of ICU admission)	1.214	1.005-1.467	0.044	1.337	1.131-1.582	0.001
Myocardial injury	2.630	0.876-7.898	0.09	2.517	0.869-7.293	0.09
Previous stroke	1.824	0.624-5.328	0.27			
Creatinine	0.999	0.995-1.003	0.58			
Lactate	1.099	0.949-1.272	0.21	1.088	0.950-1.246	0.22
ICU length of stay	0.888	0.841-0.938	<0.001	0.892	0.851-0.936	<0.001

## Discussion

In this retrospective cohort study, we examined risk factors associated with 28-day mortality in a sample of 114 patients with COVID-19 admitted to the ICU of our hospital during the first wave in the Netherlands. In our study, we found several significant differences between survivors and non-survivors, namely both groups differed regarding age, APACHE IV and SOFA score, presence of myocardial injury, previous stroke, creatinine, lactate, duration of IMV days, ICU length of stay and CRRT. In-depth analysis of these results using Cox regression demonstrated an association between higher SOFA score and 28­-day mortality. These results are in accordance with previous reports [3,16,17].

We reported a 28-day mortality of 27.2%, which is in line with previous studies performed in Italy by Graselli et al. and Spain by Barrasa et al. [3,18], as well as with the more recent COVID-ICU study by the REVA Network examining patients from Belgium, France and Switzerland [19]. However, prone ventilation was more prevalent in our report and the COVID-ICU study compared to the initial data gathered in Italy and Spain (57.0% and 70.0% versus 27% and 49%, respectively). In a study conducted by Yang et al. [17], it was shown that there was a mortality rate of 61.5% among 52 ICU patients with COVID-19. In larger series, like multiple studies from Italy, Spain and the USA, mortality rates range from approximately 22% to 61% [20]. Nadeem et al. [20] state that international differences might be explained by the fact that in some countries the pandemic arrived later. This might have given them more time to prepare, resulting in an improvement of ICU care over time for patients with COVID-19. In addition, it needs to be taken into account that regional differences and alternate hospital protocols can contribute to the differences in morbidity and mortality reported in the literature [20]. Also, observed mortality rates appear to vary among previous studies depending on the amount of patients that were already discharged at the moment of reporting [21]. Aetiologies of viral pneumonia might offer an additional explanation for the differences in mortality rate. Current data from around the world indicate that pneumonia caused by COVID-19 is much more deadly than other aetiologies like Influenza [2]. Besides this, several clinical factors appear to contribute to mortality rates, like age, SOFA score and obesity [17,20,22-24].

While analyzing the groups of survivors and non-survivors, we found that patients in the non-survivor group had a significantly older age, higher APACHE IV- and SOFA-scores on the first day of ICU admission, higher prevalence of stroke as a comorbidity and increased baseline serum levels of creatinine, lactate and hs-cTnT compared to patients in the survivor group. Previous studies examining risk factors associated with mortality in COVID-19 infection demonstrated similar results [2,16,17,24-26]. In a cohort of 733 patients, Xie et al. showed that older age, the presence of malignancies, high APACHE-II scores, high D-dimer levels and low PaO2/FiO2 levels, high creatinine levels, high hscTNI levels and low albumin levels were independent risk factors for 28-day mortality [2].

In contrast to our data, some other reports also found a significantly increased D-dimer and ferritin in deceased COVID-19 patients [19,27-29]. Hyperferritinemia is characteristic of secondary hemophagocytic lymphohistiocytosis (sHLH), a hyperinflammatory syndrome accompanied by cytopenias, multi-organ failure and cytokine release that are associated with adverse clinical outcomes [30]. Perhaps our sample size was insufficient to establish a significant difference.

Several limitations need to be taken into account. First, this study was a small sample retrospective cohort study, which might impact the generalizability of our results. Secondly, practice guidelines in our center regarding antiviral and corticosteroid therapy regimens for COVID-19 infection have been subjected to significant revision after this study’s examination period. For example, routine administration of dexamethasone to hospitalized COVID-19 patients was implemented following the publication of the RECOVERY trial [31]. Therefore, factors such as the number of patients receiving a specific therapy regimen or 28-day ICU mortality at our center might have changed after the examination period. In addition, we encountered a relatively small amount of missing data when examining patients included during this period (see Table 5, Appendix).

Lastly, it needs to be addressed that, due to the attempt to spread the COVID-19 patients equally among Dutch ICUs, the clinically stable patients were transported, leaving the potentially more severely ill patients on our ICU. Author JvO contacted all hospitals to which patients were transported to update the most important data on a weekly basis.

## Conclusions

This study showed that in our cohort of critically ill patients with COVID-19, the 28-day mortality rate was 27.2%. These patients were older, presented with a higher severity of illness at ICU admission and suffered from more organ failure. With the exception of previous stroke, there were no differences in the prevalence of comorbidity at ICU admission between survivors and non-survivors.

## Appendices

**Table 5:** The frequency of missing data of variables included in the multivariate model. Abbreviations: APACHE, Acute Physiological and Chronic Health Evaluation; SOFA, Sequential Organ Failure Assessment; CRRT, continuous renal replacement therapy; IMV, invasive mechanical ventilation.

Variables	Missing data
Age	0 (0%)
APACHE-IV (first day of ICU admission)	3 (2.6%)
SOFA (first day of ICU admission)	0 (0%)
Myocardial injury	0 (0%)
Previous stroke	0 (0%)
Creatinine	1 (0.9%)
Lactate	1 (0.9%)
Duration of IMV days	0 (0%)
CRRT	0 (0%)
ICU length of stay	0 (0%)
